# McArdle Disease: Insights Into a Rare Metabolic Myopathy in a Young Boy With Recurrent Exercise-Induced Muscle Weakness

**DOI:** 10.7759/cureus.78490

**Published:** 2025-02-04

**Authors:** Reem Abdulla A Ali, Raafat Hamad Seroor H Jadah

**Affiliations:** 1 General Practice, Bahrain Defence Force Royal Medical Services, Riffa, BHR; 2 Pediatric Neurology, Bahrain Defence Force Royal Medical Services, Riffa, BHR

**Keywords:** creatine kinase elevation, exercise intolerance, genetic diagnosis, glycogen storage disease type v, mcardle disease, metabolic myopathy, myophosphorylase deficiency, pediatric neuromuscular disorder, recurrent muscle weakness, whole-exome sequencing

## Abstract

McArdle disease, or glycogen storage disease type V, is a rare metabolic myopathy caused by a deficiency of myophosphorylase, leading to impaired glycogen breakdown in skeletal muscle. This results in exercise intolerance, muscle cramps, and episodic weakness. Due to its rarity and variable presentation, diagnosis is often delayed or misattributed to other neuromuscular disorders. We report the case of a nine-year-old boy who presented to the emergency department with acute difficulty in walking associated with left hip pain. He had a prior episode at the age of five with similar complaints, during which routine hematological and biochemical investigations, including creatine kinase (CK) levels, were unremarkable. He was initially diagnosed with viral myositis and managed conservatively. In the current episode, a physical examination revealed proximal muscle weakness with tenderness over the left hip. Laboratory investigations showed a markedly elevated CK level of 1390 IU/L, suggesting muscle pathology. Imaging studies, including hip ultrasonography and lumbosacral spine MRI, were normal. Given his clinical history and episodic muscle weakness, whole-exome sequencing was performed, which confirmed pathogenic variants in the PYGM gene, establishing the diagnosis of McArdle disease. The patient was referred to a dietitian and placed on a structured nutritional and exercise regimen, leading to significant symptomatic improvement. This case highlights the importance of considering McArdle disease in children presenting with recurrent exertional muscle weakness and myalgias, particularly in the setting of elevated CK levels. Early recognition and genetic confirmation are crucial to prevent complications and guide appropriate dietary and exercise-based interventions. Increased awareness among clinicians can facilitate timely diagnosis and improve long-term outcomes in affected individuals.

## Introduction

McArdle disease, or glycogen storage disease type V, is a rare autosomal recessive disorder caused by mutations in the PYGM gene, which encodes the muscle-specific isoform of glycogen phosphorylase. This defect impairs glycogen breakdown in skeletal muscle, leading to exercise intolerance, muscle pain, and weakness. It was first described by Brian McArdle in 1951 and has an estimated prevalence of 1 in 100,000 individuals [1]. The disease predominantly affects skeletal muscle, while other tissues, such as the liver and brain, retain normal glycogen phosphorylase activity. Clinical symptoms typically present in childhood or adolescence, with affected individuals experiencing fatigue, myalgia, and muscle cramps, often following physical exertion. Severe cases may progress to rhabdomyolysis [1,2].

The diagnosis of McArdle disease is challenging and often delayed, as symptoms can be non-specific and mimic other neuromuscular disorders. A definitive diagnosis is made through genetic testing, such as whole-exome sequencing (WES), which identifies pathogenic variants in the *PYGM* gene [2]. Laboratory findings often include elevated creatine kinase (CK) levels, reflecting muscle injury. While there is no cure for McArdle disease, management strategies focus on dietary interventions to optimize glycogen stores and tailored exercise regimens to prevent symptom exacerbation. Early diagnosis and appropriate treatment are key to improving the functional status and quality of life for affected individuals [3].

## Case presentation

A nine-year-old boy presented to the emergency department with acute difficulty in walking, which was associated with left hip pain. The pain was insidious in onset and progressively worsened over the course of a few hours. There was no history of preceding trauma, fever, or recent illness. The patient denied any weakness in the upper limbs or other parts of the lower limbs and did not report any sensory disturbances, bowel or bladder dysfunction, or systemic symptoms such as weight loss or night sweats. His previous medical history was notable for a similar episode at the age of five years, during which he experienced transient difficulty in walking with lower limb pain. At that time, he underwent a series of investigations, including routine hematological and biochemical tests, with a recorded CK level of 97 IU/L, which was within normal limits. He was admitted with a presumptive diagnosis of viral myositis and was managed conservatively with symptomatic treatment, including antipyretics and analgesics. His symptoms resolved, and he was subsequently discharged home without further follow-up.

His perinatal history was uneventful. He was born at full term via spontaneous vaginal delivery at 38 weeks of gestation, with no history of neonatal intensive care unit admission. Growth and developmental milestones were appropriate for age, and there was no reported history of delayed motor development or difficulties in physical activities. Regarding his family history, there was no evidence of hereditary or metabolic disorders, and no family members had been diagnosed with muscular diseases. His parents were non-consanguineous, and there was no history of similar presentations among siblings or extended relatives.

Upon examination, the patient was found to be alert, oriented, and in no apparent distress. His vital signs were stable, with a heart rate of 92 beats per minute, blood pressure of 110/70 mmHg, respiratory rate of 18 breaths per minute, and oxygen saturation of 99% on room air. Neurological examination revealed normal cognitive function and speech. Motor system examination showed normal muscle tone in all limbs, with no signs of atrophy or fasciculations. Muscle power was graded 4 out of 5 in the proximal lower limbs, particularly in the hip flexors and extensors, while the distal limb strength remained preserved. Deep tendon reflexes were intact and symmetrical, and there were no pathological reflexes. Sensory examination was normal. The patient exhibited tenderness on palpation of the left hip, with associated pain during passive and active movements. He was unable to bear weight or ambulate independently. The remainder of the systemic examination, including cardiovascular, respiratory, and abdominal assessments, was unremarkable.

Given the clinical presentation and history of recurrent lower limb weakness, an initial diagnosis of a neuromuscular disorder, including muscular dystrophy, was considered. A comprehensive work-up was undertaken. Laboratory investigations revealed a markedly elevated serum CK level of 1390 IU/L, suggestive of muscle injury. Other routine hematological and biochemical parameters, including complete blood count, liver function tests, renal function tests, and inflammatory markers, were within normal limits. Urinalysis showed no evidence of myoglobinuria. Imaging studies, including ultrasonography of the hip and magnetic resonance imaging of the lumbosacral spine, were performed and reported as normal, ruling out structural abnormalities or inflammatory myopathies.

Given the episodic nature of his symptoms and the elevated CK levels, an inherited metabolic myopathy was suspected. WES was performed, which identified pathogenic variants in the PYGM gene (1176C>T and 855+1G>A), confirming the diagnosis of glycogen storage disease type V (McArdle disease). Following the confirmation of his diagnosis, the patient was referred to a dietitian for tailored nutritional management. He was started on a specific diet aimed at optimizing carbohydrate intake to improve energy metabolism and prevent exercise-induced symptoms. Over the course of follow-up, he demonstrated significant improvement in his functional status, with a reduction in episodes of exertional weakness and pain. He and his family were educated on strategies to manage his condition, including avoiding prolonged anaerobic activity, ensuring adequate pre-exercise carbohydrate intake, and adopting graded aerobic exercise training. The patient was scheduled for regular follow-up to monitor his progress and adjust management strategies as needed.

## Discussion

McArdle disease is a rare genetic disorder classified as a glycogen storage disease, caused by deficient myophosphorylase activity. Myophosphorylase is a key enzyme in energy metabolism, as it catalyzes the rate-limiting step in glycogenolysis in muscle [4,5]. Glycogen phosphorylase has three isoforms: PYGB (brain), PYGL (liver), and PYGM (muscle). While PYGM is predominantly found in skeletal muscle, it is also present in other tissues and organs, though its role outside of muscle remains unclear. It is presumed that PYGM may contribute not only to McArdle disease but also to other conditions [6]. To date, around 150 mutations in the human PYGM gene have been identified, with the p.R50X nonsense mutation in exon 1 being the most prevalent in Caucasians with McArdle disease [3]. By contrast, McArdle disease is extremely rare in East Asia. For example, only one case has been reported in Japan, and despite a global prevalence of approximately 1 in 100,000 individuals, more than 50% of PYGM mutations in Europe are caused by the p.R50X mutation [7]. This has led to the hypothesis that there are ethnic differences in the genetic variation of PYGM between European and Asian populations. Additionally, studies show that 54% of PYGM mutations are homozygous, with the remainder being heterozygous [8].

The hallmark symptom of McArdle disease is exercise intolerance, which is often accompanied by painful muscle cramps, weakness, and fatigue. Muscle pain and stiffness can sometimes result in contractures, particularly following the onset of activity. Symptoms are most pronounced early in exercise but improve with cessation. In cases of high-intensity exercise, sudden, persistent muscle contractions can lead to severe muscle damage, resulting in the massive release of muscle proteins, including creatine kinase (typically >1,000 IU/L) and myoglobin, leading to myoglobinuria (dark-colored urine). Rhabdomyolysis, a severe form of muscle breakdown, can lead to acute renal failure and potentially life-threatening hyperkalemia [2].

A unique phenomenon associated with McArdle disease is the "second-wind" effect, where many patients experience symptom relief after about 10 minutes of gentle aerobic activity [3]. Despite these challenges, most individuals with McArdle disease lead a normal life, with no impact on life expectancy. However, rhabdomyolysis should be avoided, as it can result in acute renal failure. In a small subset of patients, there may be a progressive worsening of symptoms with advancing age, particularly affecting the shoulder girdle and back muscles [4].

Treatment primarily involves lifestyle and dietary modifications. Studies have shown that a carbohydrate-rich diet leads to significantly better outcomes compared to a protein-rich diet, as it helps improve muscle energy availability during physical activity [9].

## Conclusions

McArdle disease is an extremely rare but significant metabolic disorder, primarily presenting with exercise-induced muscle pain, weakness, and fatigue. Early diagnosis, particularly through WES, is crucial for accurate identification and management. Treatment strategies, including dietary modifications, with a focus on a carbohydrate-rich diet, offer substantial improvements in patient outcomes compared to protein-rich diets. Early intervention and ongoing management are essential to improving functional status and quality of life in affected individuals.
